# The familial dysautonomia disease gene *IKBKAP* is required in the developing and adult mouse central nervous system

**DOI:** 10.1242/dmm.028258

**Published:** 2017-05-01

**Authors:** Marta Chaverra, Lynn George, Marc Mergy, Hannah Waller, Katharine Kujawa, Connor Murnion, Ezekiel Sharples, Julian Thorne, Nathaniel Podgajny, Andrea Grindeland, Yumi Ueki, Steven Eiger, Cassie Cusick, A. Michael Babcock, George A. Carlson, Frances Lefcort

**Affiliations:** 1 Department of Cell Biology and Neuroscience, Montana State University, Bozeman, MT 59717, USA; 2 Department of Biological and Physical Sciences, Montana State University Billings, Billings, MT 59101, USA; 3 Department of Psychology, Montana State University, Bozeman, MT 59717, USA; 4 University of Washington, School of Medicine, Seattle, WA 98195, USA; 5 McLaughlin Research Institute, Great Falls, MT 59405, USA

**Keywords:** Familial dysautonomia, Elongator, Hereditary sensory and autonomic neuropathy

## Abstract

Hereditary sensory and autonomic neuropathies (HSANs) are a genetically and clinically diverse group of disorders defined by peripheral nervous system (PNS) dysfunction. HSAN type III, known as familial dysautonomia (FD), results from a single base mutation in the gene *IKBKAP* that encodes a scaffolding unit (ELP1) for a multi-subunit complex known as Elongator. Since mutations in other Elongator subunits (ELP2 to ELP4) are associated with central nervous system (CNS) disorders, the goal of this study was to investigate a potential requirement for *Ikbkap* in the CNS of mice*.* The sensory and autonomic pathophysiology of FD is fatal, with the majority of patients dying by age 40. While signs and pathology of FD have been noted in the CNS, the clinical and research focus has been on the sensory and autonomic dysfunction, and no genetic model studies have investigated the requirement for *Ikbkap* in the CNS. Here, we report, using a novel mouse line in which *Ikbkap* is deleted solely in the nervous system, that not only is *Ikbkap* widely expressed in the embryonic and adult CNS, but its deletion perturbs both the development of cortical neurons and their survival in adulthood. Primary cilia in embryonic cortical apical progenitors and motile cilia in adult ependymal cells are reduced in number and disorganized. Furthermore, we report that, in the adult CNS, both autonomic and non-autonomic neuronal populations require *Ikbkap* for survival, including spinal motor and cortical neurons. In addition, the mice developed kyphoscoliosis, an FD hallmark, indicating its neuropathic etiology. Ultimately, these perturbations manifest in a developmental and progressive neurodegenerative condition that includes impairments in learning and memory. Collectively, these data reveal an essential function for *Ikbkap* that extends beyond the peripheral nervous system to CNS development and function. With the identification of discrete CNS cell types and structures that depend on *Ikbkap*, novel strategies to thwart the progressive demise of CNS neurons in FD can be developed.

## Abstract (precis)

**Summary:** *Ikbkap* is essential for normal CNS development, neuronal survival and behavior, adding to our understanding of the role of the Elongator complex in the mammalian CNS.

## INTRODUCTION

There are seven types of hereditary sensory and autonomic neuropathies (HSANs) that result from mutations in 16 different genes (Auer-Grumbach, 2013; Tourtellotte, 2016). HSAN type III, or familial dysautonomia (FD; also called Riley Day syndrome) results from a point mutation (IVS20+6T>C mutation) in the gene *IKBKAP* (Anderson et al., 2001; Slaugenhaupt et al., 2001). The mutation is in a splice donor site that prevents the normal inclusion of exon 20, causing a frame shift, which ultimately leads to production of an mRNA that is degraded by nonsense-mediated decay (Boone et al., 2012). *IKBKAP* is widely expressed, but cell types vary in their ability to splice the mutated message, with neurons being the least capable of producing a normal *IKBKAP* mRNA (Cuajungco et al., 2003). Thus, patients vary in their phenotype, and most cells contain a mixture of wild-type and mutant mRNA (Cuajungco et al., 2003). This leads to reductions in the encoded protein, IKAP (or ELP1), in the central and peripheral nervous systems (CNS and PNS, respectively). Classic hallmarks of the disease include decreased pain and temperature sensation, orthostatic hypotension, kyphoscoliosis and dysautonomic ‘crises’ marked by vomiting, erratic hypertension and tachycardia (Axelrod, 2006; Norcliffe-Kaufmann et al., 2016; Dietrich and Dragatsis, 2016).

Numerous functions for IKAP have been proposed, including critical roles as part of the six subunit Elongator complex in both the nucleus (Hawkes et al., 2002; Kim et al., 2002; Svejstrup, 2007) and cytoplasm. Accruing evidence now demonstrates that Elongator is required for tRNA modification, histone acetylation, actin organization and exocytosis (Rahl et al., 2005; Esberg et al., 2006; Karlsborn et al., 2014b; Tielens et al., 2016) in organisms ranging from yeast to mammals. Studies have demonstrated that tRNA from FD patients contains reduced levels of the modified wobble nucleoside mcm^5^s^2^U_34_ (Lin et al., 2013; Karlsborn et al., 2014a; Yoshida et al., 2015), lending support to a direct function of IKAP in translation. Interestingly, three other Elongator subunits, *ELP2* to *ELP4*, have each been associated with CNS disorders (Nguyen et al., 2010): *ELP2* and *ELP4* have been implicated in intellectual development disorders (Cohen et al., 2015; Franic et al., 2015; Addis et al., 2015) and epilepsy (Gkampeta et al., 2014; Reinthaler et al., 2014; Strug et al., 2009), and *ELP3* with amyotrophic lateral sclerosis (Simpson et al., 2009; Kwee et al., 2012).

Since disruptions to autonomic functions are the most life-threatening feature of FD, and the fact that FD is classified as one of the HSANs, the majority of FD research has focused on the sensory and autonomic nervous system (ANS), which are devastated in humans (Norcliffe-Kaufmann et al., 2016; Dietrich and Dragatsis, 2016; Tourtellotte, 2016) and mouse models (Dietrich et al., 2012; George et al., 2013; Jackson et al., 2014; Morini et al., 2016). Mice that are completely null for *Ikbkap* die by embryonic day (E)10 due to a failure in neurulation and vasculogenesis (Chen et al., 2009; Dietrich et al., 2011). Mouse models have revealed that the neuronal loss in the PNS is not due to failure in neural crest migration – the precursor cells that give rise to the majority of the peripheral nervous system – but rather to the apoptotic death of the sensory progenitors and neurons in the embryonic sensory and sympathetic ganglia (George et al., 2013; Jackson et al., 2014). Importantly, clinical studies have indicated that the CNS is also disrupted in FD. Clinical symptoms involving the CNS include visual impairment (Mendoza-Santiesteban et al., 2012, 2014; Ueki et al., 2016), high anxiety levels, learning impairments (Day, 1979; Sak et al., 1967; Welton et al., 1979; Clayson et al., 1980; Axelrod et al., 2012; Norcliffe-Kaufmann et al., 2016), seizures, decreased motor nerve conduction, deficits in brain stem reflexes (Gutiérrez et al., 2015) and abnormal electroencephalogram (EEG) activity (Niedermeyer et al., 1967; Ochoa, 2003). Neuroimaging studies [magnetic resonance imaging (MRI) and diffusion tensor imaging (DTI)] demonstrate both white and gray matter microstructural damage in FD brains (Axelrod et al., 2010), and pathological studies have revealed enlarged 4th ventricles associated with atrophy in the medulla (Engel and Aring, 1945; Cohen and Solomon, 1955; Brown et al., 1964). Most patients die due to sudden death during sleep or respiratory failure (Axelrod, 2006). Given that the ANS is composed of neurons of both the CNS and PNS, it would not be surprising if deficits in FD could also result from disruptions in the central components of the ANS (Saper, 2002).

The goal of this study was to investigate a potential function for *Ikbkap* in the CNS. To this end, we generated mice in which *Ikbkap* is deleted in the CNS, beginning in early development. We report here extensive deficits in both the development and maintenance of the CNS, and a requirement for *Ikbkap* in several CNS neuronal populations, both within and outside of the ANS, including the cortex, brainstem nuclei and spinal motor neurons. Although *Ikbkap* is expressed widely throughout the body – including in several internal organs – selective deletion of *Ikbkap* in the nervous system is sufficient to recapitulate several FD hallmarks, including kyphoscoliosis. These data not only complement recent studies that implicate a key role for another Elongator subunit, *Elp3*, in cortical development (Creppe et al., 2009; Laguesse et al., 2015; Tielens et al., 2016) but also expand our understanding of roles served by the Elongator complex in CNS development, maintenance and behavior.

## RESULTS

**Fig. 1.**
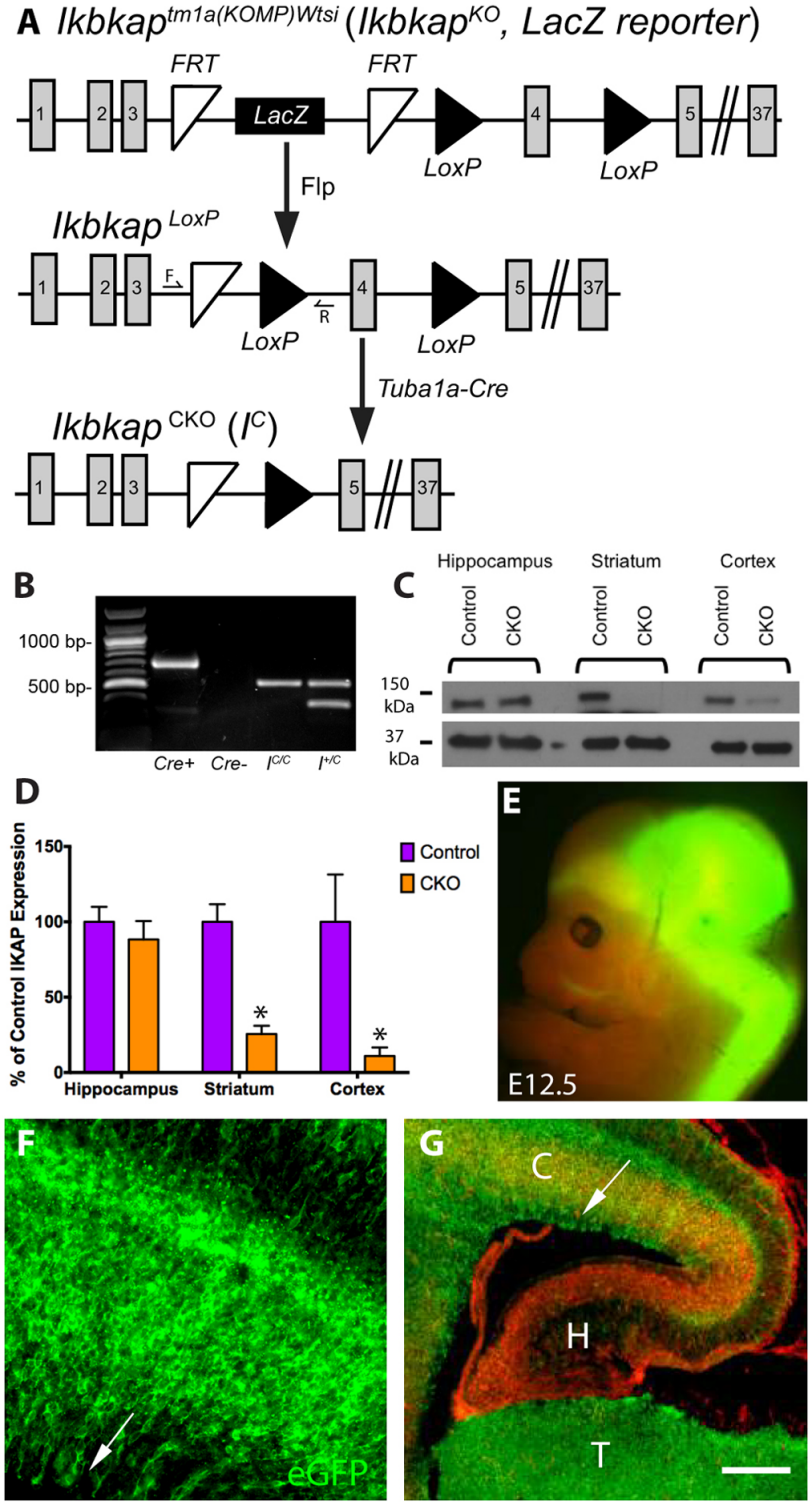
**Generation of *Tuba1a-Cre;Ikbkap* CKO mice and expression *Tuba1a-cre* in the developing CNS.** (A) Schematic of conditional mutagenesis in the *Ikbkap* gene. The β-gal cassette in the knockout *Ikbkap^tm1a(KOMP)Wtsi^* allele, which does not express *Ikbkap*, was removed via Flippase-mediated recombination. In the new *Ikbkap^LoxP^* allele, *Ikbkap* is expressed, but exon 4 is flanked by LoxP sites (black triangles) and is excised by Cre in cells that express *Tuba* in *Tuba1a-Cre;Ikbkap^LoxP/LoxP^* mice. F and R indicate the locations of primers (forward and reverse, respectively) used to distinguish the CKO *Ikbkap* allele from the wild-type allele. (B) PCR genotyping. (C,D) Western blots on control and CKO hippocampus, striatum and cortex, and quantification of protein intensity by densitometry demonstrate that IKAP protein is reduced in the striatum and cortex but not in the hippocampus. (E-G) Crossing *ROSA^mT-mG/mT-mG^* mice to the *Tuba1a-Cre* mice reports expression of the *Tuba1a-Cre* at (E) E12.5 throughout the brain and spinal cord, with detectable expression in the trigeminal nerve, (F) expression in the E17.5 cortex in both the ventricular, subventricular zone and neuronal layers, and expressed in the (G) E17.5 thalamus, but not in the hippocampus. GFP reports *Cre* expression; Tomato is expressed where *Cre* is inactive. Arrows in F and G point to the ventricular zone of the cortex; ‘C’, cortex; H, hippocampus; T, thalamus. Scale bars: 1 mm (E); 100 μm (F); 450 μm (G).

The goal of this study was to investigate a role for *Ikbkap* in the CNS given the fact that CNS disruptions have been noted in FD patients. To that end, we obtained a *Tuba1a-Cre* mouse line in which Cre activity is specific to the PNS and CNS and in which Cre expression is initiated by E11, and crossed it to our floxed *Ikbkap* line to generate *Ikbkap* conditional knockout (CKO) mice (Fig. 1A,B) (Gloster et al., 1999; Coppola et al., 2004; George et al., 2013). Western blots demonstrate reduced IKAP protein levels in the cortex and striatum of *Tuba1a-Cre; I^C/C^* mice (Fig. 1C,D). To determine the structures and cell types in which deletion occurred, we crossed the *Tuba1a-Cre* mice to *ROSA^mT-mG/mT-mG^* Cre reporter mice. GFP expression revealed Cre activity in the brain, brainstem and spinal cord at E12.5 (earliest time point examined; Fig. 1E), and in the embryonic cortex in the ventricular and intermediates zones, as well as in the cortical plate at E17.5 (Fig. 1F). Surprisingly, while strong Cre activity was detected in the E17.5 cortex and thalamus, it was not detected in the hippocampus (Fig. 1G). Cre expression was also absent in older postnatal hippocampus (1 month) (Fig. S1a). Western blot analysis in the adult CKO brain confirms the structure-specific Cre activity, where IKAP protein was reduced in the cortex and striatum but not in the hippocampus (Fig. 1C,D).

While examination of live unfixed embryonic and adult tissue revealed robust Cre expression in the CNS, it was only upon fixation and staining with antibody that we could visualize Cre expression in the embryonic or adult PNS. We sought to determine the subsets and percentage of peripheral neurons in which Cre was active in the dorsal root ganglia (DRG) and sympathetic ganglion (Fig. S1). In 1-month-old mice, approximately 48.5% (±4.2 s.d.; *n*=3 embryos) of small diameter Substance P-positive nociceptors, 47.5% (±10.6 s.d.; *n*=3 embryos) of CGRP-positive small- and medium-sized neurons, and 22.5% (±6.0 s.d.; *n*=3 embryos) of large diameter proprioceptors (TrkC positive) were targeted by Cre in the DRG, and 31.6% (+6.5; *n*=2 embryos) of the tyrosine hydroxylase-positive cells in the sympathetic ganglia chain expressed Cre. In the brain, besides its expression in neurons and neural progenitors, *Tuba1a-Cre* was expressed in approximately half of the Olig2-positive glial progenitors in the subventricular zone (Fig. S1g-l). Given that FD patient neurons are heterogeneous in their expression levels of IKAP, this new mouse line provides a useful model system in which to dissect the pathophysiological mechanisms mediating some of the hallmark features of the human disease.

**Fig. 2.**
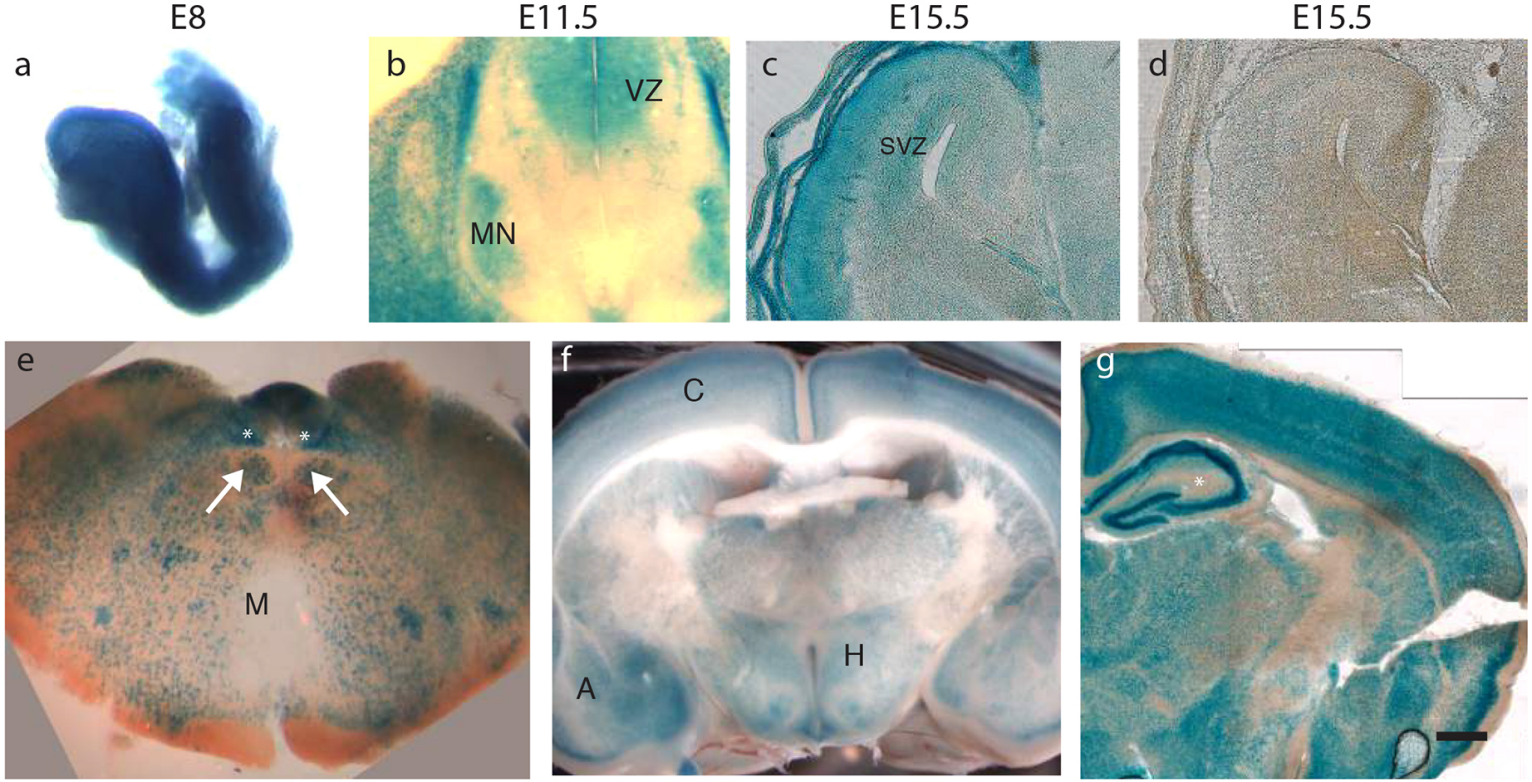
***Ikbkap* expression in developing and adult CNS.** *Ikbkap:LacZ* reporter mouse shows β-gal expression (A) throughout the neural tube at E8, (B) in the spinal cord motor neuron columns (MN) and ventricular zone (VZ) at E11.5, (C) in both the VZ and subventricular zone (SVZ) and cortical plate in the embryonic cortex at E15.5, with a control shown for comparison in (D), and in (E) several nuclei in the adult medulla (M), including the nucleus tract solitarius (NTS) (*) and cranial motor nuclei (arrows), and (F,G) in the adult cortex (‘C’), hypothalamus (H) and amygdala (‘A’) and hippocampus (* in G). Scale bars: 200 µm (D); 500 µm (E,F).

To obtain the necessary background information in order to interpret the CKO phenotype, we first determined which structures and cell types expressed *Ikbkap*; such information is lacking for human tissues. To do so, we analyzed β-galactosidase (β-gal) expression in *Ikbkap:LacZ* reporter mice (George et al., 2013). At E8, β-gal was expressed throughout the neural tube (Fig. 2A), which may explain why embryos that are completely null for *Ikbkap* fail to complete neurulation (Chen et al., 2009; Dietrich et al., 2011). By E11.5, *Ikbkap* expression was detected in the ventricular zone of the spinal cord and in spinal motor neurons (Fig. 2B). At E15.5, *Ikbkap* was expressed in the mitotic and post-mitotic zones of the developing cortex and in the choroid plexus (Fig. 2C,D). In the adult, *Ikbkap* was expressed throughout the brain, including in the medulla, cortex, amygdala, hippocampus and hypothalamus (Fig. 2E-G). Importantly, these data demonstrate that *Ikbkap* is broadly expressed during CNS development and in the adult brain, and is expressed in key nuclei in the adult brain and brainstem that regulate the ANS. These data also reveal *Ikbkap* expression in the ventricular and subventricular zones of the cortex, the sites of cortical and spinal neurogenesis. Finally, assuming *IKBKAP* is also expressed in human motor neurons, the robust *Ikbkap:LacZ* activity observed in spinal motor neurons was of interest given the underlying gait disruption and spinal deformities exhibited by FD patients.

### Reduction of IKAP in the CNS induces deficits that are observed in FD

**Fig. 3.**
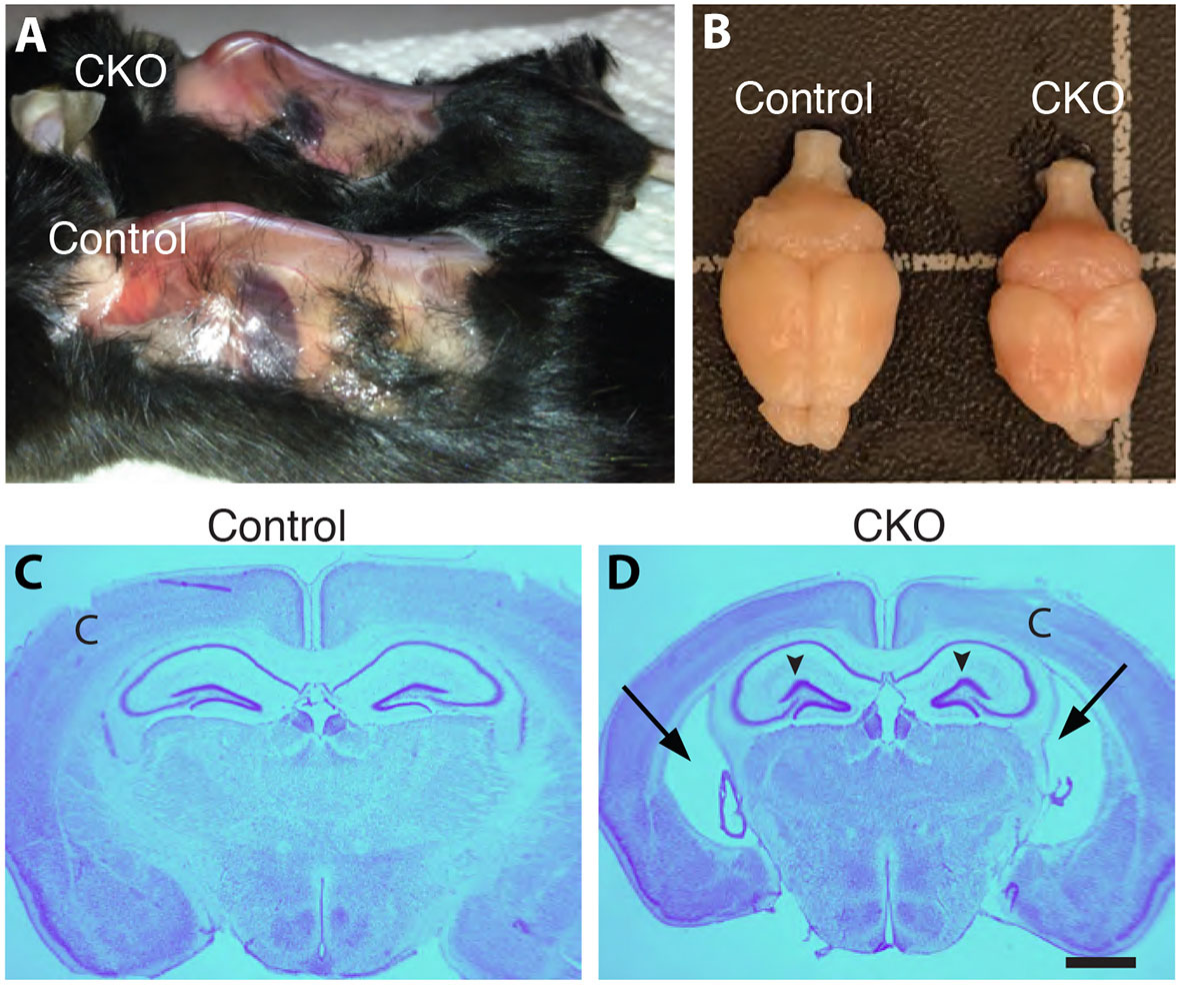
**Phenotype of *Tuba1a-Cre;I^C/C^* mice.** (A) CKO mice exhibit kyphosis and (B) microcephaly. (C,D) Nissl staining of coronal sections indicates CKO mice have enlarged lateral ventricles (arrows) and rounded hippocampi with a distorted dentate gyrus lateral blade (arrowheads). Scale bar: 500 µm (D, applies to C). ‘C’, cortex.

*I^C/C^* mice grow normally while littermate *Tuba1a-Cre;I^C/C^* CKO mice develop a progressive degenerative condition that shares some of the classic hallmarks of human FD: they are smaller than their control littermates, develop kyphosis, and have a slow and unsteady gait (Movies 1 and 2; Fig. 3A), with approximately 50% of CKO mice dying by 5 months of age, often suddenly. The weight of CKO mice was also significantly reduced compared to their control littermates (Table 1), and this difference was apparent by 3 weeks (earliest time point measured), becoming more pronounced with age. CKO mice also exhibited a rough dull coat. When picked up by their tail, control mice extended their hind limbs laterally, while CKO mice exhibited hind limb (and often forelimb) clasping (Movie 3; Table 1), an indication of disruption in descending CNS motor tracts (Guyenet et al., 2010). Analyses of motor behaviors in CKO mice and controls indicated diminished bar balance, reduced grip strength, dysmetria, an abnormal gait and a tremor in the CKO, but normal response to toe pinching and hot temperatures (Table 1). Given that only 50% of the nociceptors were targeted by the Cre, a normal response to noxious stimuli is not unexpected. Motor problems could be the consequences of deletion of *Ikbkap* from upper and/or lower motor neurons, brainstem nuclei and extrapyramidal neurons, all sites of *Ikbkap* and Cre-expression.

**Table 1.**
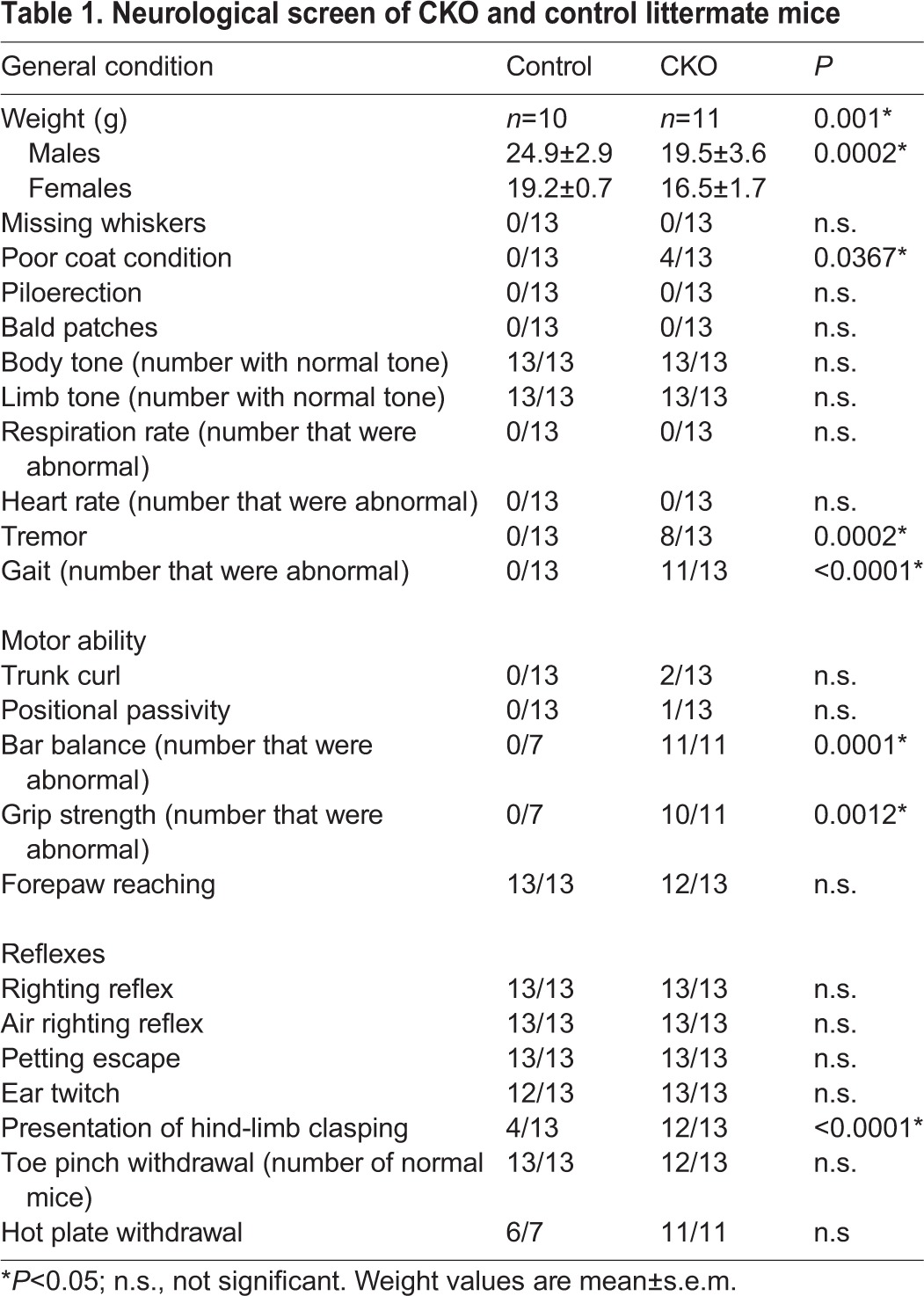
**Neurological screen of CKO and control littermate mice**

Morphological analyses of the brains of adult CKO mice indicated that they are reduced in size but otherwise grossly normal (Table 2; Fig. 3B). Nissl staining on coronal sections indicated that the cortex is layered normally, but the lateral ventricles were typically enlarged (Fig. 3C,D; Table 2), an observation confirmed by quantification of the area occupied by the lateral ventricles relative to the hemisphere area (Table 2). In addition, in the CKO brain, the corpus callosum and the lateral amygdaloid nucleus in the CKO brain were significantly reduced, while the hippocampus was relatively enlarged with respect to the reduced hemisphere (Table 2), which we attribute to the absence of Cre expression in the hippocampus (Fig. 1G; Fig. S1a,b).

**Table 2.**
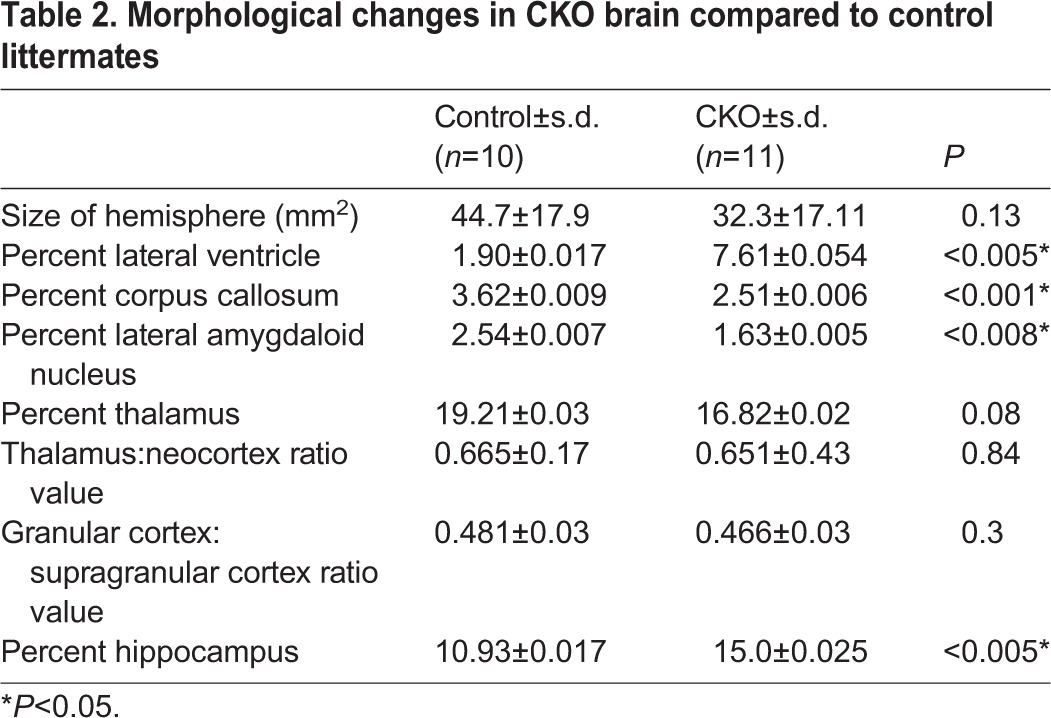
**Morphological changes in CKO brain compared to control littermates**

**Fig. 4.**
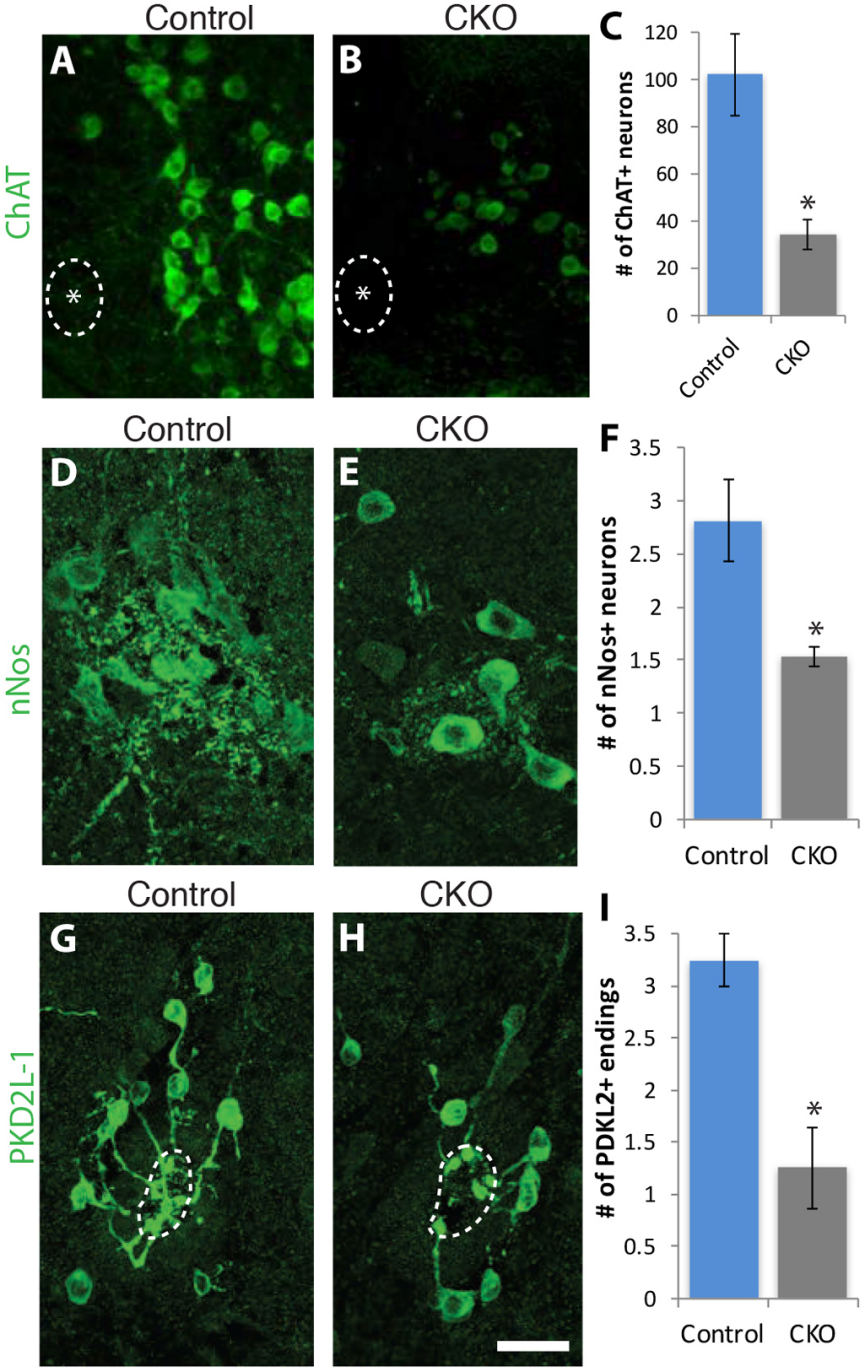
**CNS autonomic nuclei neuron number is reduced in the absence of *Ikbkap*.** (A-C) Motor neurons (ChAT positive) in the DMV are reduced in the CKO (see also Table 2). (D-F) nNOS-positive neurons that comprise the intermediolateral cell columns are significantly reduced in number in the CKO thoracic spinal cord. (G-I) PKD2L1-positive cells extend intraluminal buds into the central canal of the spinal cord and are reduced in number and complexity compared to controls. Dashed lines, central canal of spinal cord. Asterisks in A and B denote midline. Error bars denote s.e.m. *P<0.05, unpaired Student's *t*-test (C,F,I). Scale bar: 30 µm (A,B); 10 µm (D,E,); 20 µm (G,H).

Given the critical role that CNS regulatory structures play in ANS output, we examined three different ANS neuronal populations in the CNS. Because human FD pathology studies show reductions in the dorsal vagal motor nucleus (DVM) (Engel and Aring, 1945; Dyck et al., 1978) and in the intermediolateral (IML) cell column, we investigated the integrity of those populations in mutant and control mice (Fig. 4). To visualize the DMV cells, we immunostained sections with antibodies against choline acetyltransferase (ChAT), and to visualize the intermediolateral cell column, we stained with antibodies against neuronal nitric oxide synthase (nNOS). Both of these motor neuron populations were significantly reduced in the CKO mice with respect to their control littermates (Fig. 4A-F). Another important population of neurons in the spinal cord that contributes key afferent signals to the ANS are the PKD2L1-positive cells in the subependymal zone surrounding the central canal that directly contact cerebral spinal fluid (Huang et al., 2006; Djenoune et al., 2014; Orts-Del'Immagine et al., 2014). PKD2L1 is a six-transmembrane protein (member of the transient receptor potential family) that forms a Ca^2+^ channel in primary cilia (Orts-Del'immagine et al., 2012; DeCaen et al., 2013). It is regulated by pH and osmolarity, and relays either chemical or possibly mechanical (flow) information regarding cerebral spinal fluid (CSF). Quantification of the number of PKD2L1-positive cells surrounding the central canal and the number of PKD2L1-positive cells with endings inside the central canal demonstrated a significant reduction in the CKO mouse compared to control littermates (Fig. 4G,I). Not only was the number of PKD2L1-positive cells reduced, but the morphology of their intraluminal buds in the central canal was altered: their morphology was less elaborate and they were less likely to be juxtaposed to one another than they were in the controls (Fig. 4G-I). Compared to controls, more sections from CKO mice were completely devoid of PKD2L1-positive cells that either surrounded the central canal and/or extended into the canal (although these were not significantly different), consistent with their overall number being reduced.

### *Ikbkap* is required for maintenance of muscle innervation, and for survival of spinal motor neurons and cortical neurons

**Fig. 5.**
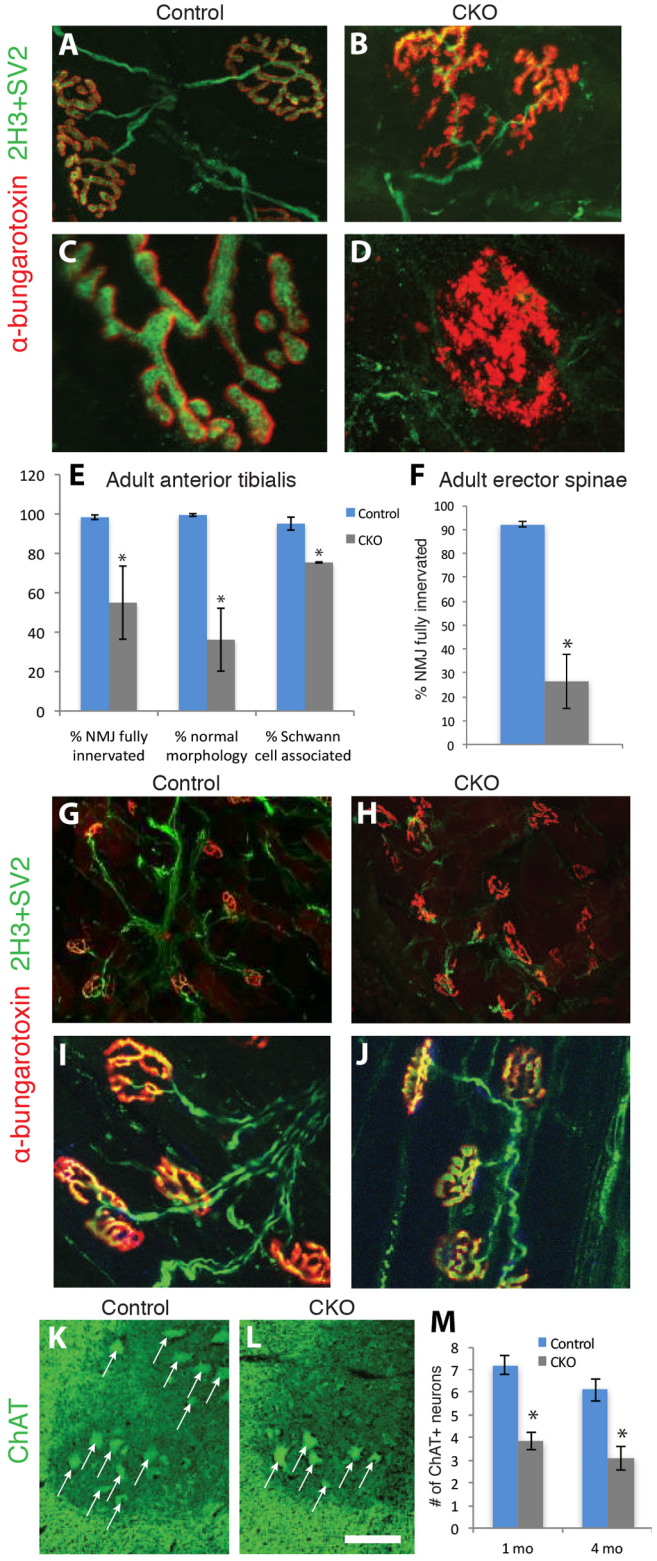
**Neuromuscular junctions in two different muscle groups are denervated in the absence of *Ikbkap*, and motor neuron numbers are reduced in the CKO spinal cord.** Adult (4 month) anterior tibialis muscles (A-E) and erector spinae muscles (F-J) were immunolabeled with α-bungarotoxin (red) to identify cholinergic receptors, and with SV2and 2H3 (green) to label synaptic endings and axons (neurofilaments), respectively. In both muscle types, there was a significant reduction in NMJ innervation, with many terminals being partially innervated (B,H) or completely denervated (D,H). In contrast, at 1 month, neuromuscular junctions are innervated properly in both CKO mice (J) and littermate controls (I). Error bars denote s.e.m. and *P-*values correspond to an unpaired Student's *t*-test (*P<0.05). Motor neuron (arrows) numbers (ChAT positive) are reduced in the spinal cords of CKO mice compared to in littermate controls at both 1 month and 4 months. Error bars denote s.e.m. and *P-*values correspond to an unpaired Student's *t*-test (*P<0.05). Scale bar: 5 µm (A,B); 2 µm (C,D); 5 µm (G,H); 15 µm (I,J); 20 μm (K,L).

Progressive gait unsteadiness is a common feature of FD (Axelrod et al., 2012) and becomes progressively more pronounced in the CKO mice as they age. For the first 2-3 months, there were no striking differences in motor agility between CKO and control mice, but after 3 months, the CKOs developed a slower, often wide-based and unsteady gait, which could develop into full hind limb weakness and dragging (Movie 2). By this age, when picked up by the tail, mutant mice exhibited hind limb clasping (Movie 3). Since in this mouse model *Ikbkap* is deleted from only approximately 20% of proprioceptors, none of which died in the embryo (Fig. S2) nor in the adult (see below) and our expression analysis demonstrated that spinal motor neurons express *Ikbkap*, we asked whether the progressive motor weakness could be due to disruptions at the neuromuscular junction (NMJ) in the CKO hind limb. Quantification of NMJ innervation of the anterior tibialis muscle revealed a striking reduction in innervated junctions and a high frequency of dysmorphic junctions: the edges were often frayed and acetylcholine receptors were diffusely distributed, a phenomenon not observed in controls (Fig. 5A-E). Although the majority of NMJs in the mutant were associated with Schwann cells, there was a significant reduction in the number of innervating axons associated with Schwann cells (Fig. 5E).

Spinal deformities are another common feature of FD: either scoliosis or kyphosis develops in >80% of FD patients (Bar-On et al., 2000; Hayek et al., 2000). Strikingly, the majority of the *Ikbkap* CKO mice become kyphotic by 4-5 months (Fig. 3A). Since kyphosis can result from neuromuscular disruptions, we quantified NMJ innervation in the erector spinae (paraspinal) muscles, which are lateral to the vertebral column and function to straighten the back (Fig. 5F-H). As in the anterior tibialis muscle, we found a significant reduction in the number of fully innervated NMJ in the CKO erector spinae muscles compared to controls (Fig. 5F). Since the weakness and kyphosis take several months to develop in CKO mice, we examined NMJ morphology and innervation in young mice to determine whether the adult phenotype was the result of a degenerative process. Mice at 1 month of age had more normal NMJ innervation and morphology (Fig. 5I,J) indicating that the NMJ progressively degenerates in the absence of *Ikbkap*.

To determine if the degeneration of NMJ innervation was associated with a reduction in the number of spinal motor neurons, we quantified the number of ChAT-positive spinal motor neurons in 4-month and 1-month mutant and control spinal cord (Fig. 5K-M). We found significantly fewer motor neurons in the CKO spinal cord compared to controls at both ages, indicating that this loss occurs early. These data suggest that juvenile CKO mice can compensate for the reduced number of motor neurons, but as they mature, their progressive weakness is due at least in part to degeneration of NMJs.

Given the progressive neurodegeneration exhibited by the CKO mice as they age, we analyzed adult *Ikbkap* CKO brains for signs of cell death. While terminal deoxynucleotidyl transferase dUTP nick end labeling (TUNEL)-positive cells were detected in both the control and CKO adult brains, there was dramatically more TUNEL expression throughout the CKO cortex than in the control cortex (Fig. S2a-c). This degeneration was not accompanied by GFAP-positive inflammation (data not shown).

### Abnormal CNS development in the absence of *Ikbkap*

**Fig. 6.**
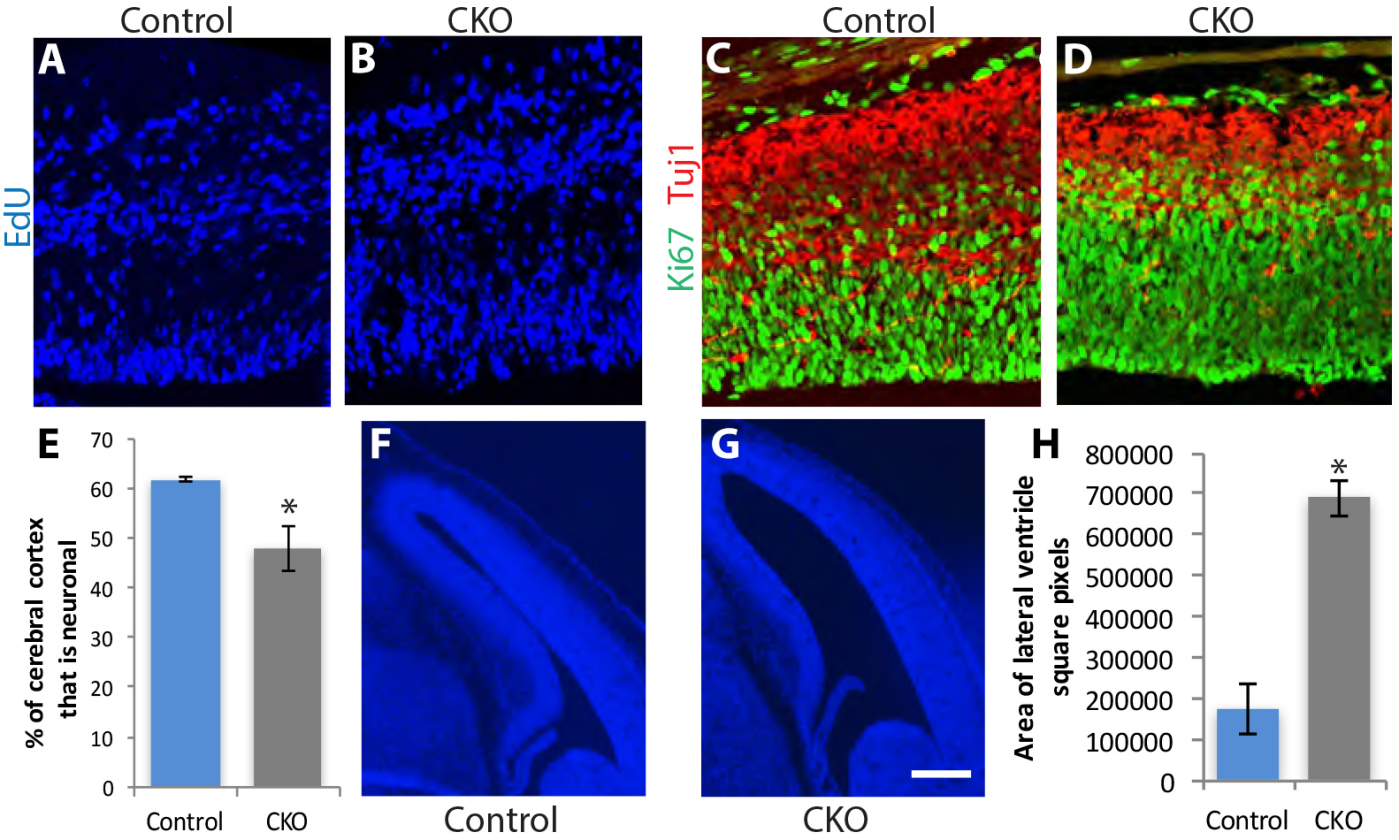
**CNS development requires *Ikbkap.*** (A,B) A 24-h pulse of EdU in E13.5 embryos indicated that progenitor cells produced daughters that migrated in a grossly normal manner to the intermediate zone and cortical plate. (C-E) Immunolabeling at E14.5 for Ki67 and Tuj1 demonstrates the presence of both progenitor cells and neurons, but that the region of the cortex occupied by neurons is thinner in the CKO than in the control brain (*P<0.038; *n*=3 embryos; Student's *t*-test). (F-H) In the absence of *Ikbkap*, the lateral ventricles are expanded in size compared to in control littermates (*n*=3 embryos; *P<0.002). Scale bar: 50 μm (A-D); 300 μm (F,G).

In addition to increased cell death contributing to the reductions in neuronal populations in the adult CKO CNS, we asked whether CNS development was impaired in the absence of *Ikbkap*. Our previous work has revealed a critical role for *Ikbkap* in the development of the peripheral nervous system (George et al., 2013). To this end, we analyzed embryonic brains at E14.5 and at E17.5 in *Tuba1a-Cre* mice crossed to the *ROSA^mT-mG/mT-mG^* strain. These Cre reporter analyses showed that Cre was strongly active in the brain by E12.5 (earliest age examined), including expression in mitotically active progenitors in the ventricular and subventricular zone of the cortex, as well as in post-mitotic neurons. *Ikbkap* was also expressed in these same cell populations during the same time frame (Fig. 2). To determine the requirement for *Ikbkap* during cortical neurogenesis, we pulsed pregnant dams with EdU at E13.5 and harvested the embryos 24 h later. EdU staining indicated that EdU-positive neurons were generated in the ventricular and subventricular zones, and migrated away forming a morphologically normal intermediate zone and cortical plate (Fig. 6A,B). Similarly, Ki67-positive progenitors were prevalent in the ventricular and subventricular zone (Fig. 6C,D). However, two abnormalities were evident in the *Ikbkap* CKO brain: (1) the cortical plate and subplate, relative to the cortex as a whole, were significantly thinner (less Tuji1-positive immunoreactivity) in the CKO brain compared to their control littermates (Fig. 6E) and (2) the lateral ventricles were significantly enlarged at both E14.5 (Fig. 6F-H) and E17.5 (52% increase; *n*=3 embryos; *P*<0.036), with the cortical hemispheres expanded in size relative to the developing thalamus. To determine if the reduction in neuronal numbers was due to apoptosis, we assessed the expression of TUNEL at both E14.5 and E17.5 and saw no difference in levels between the mutant and control cortex (Fig. S2d-e). This result was surprising because when *Ikbkap* was deleted from the neural crest using a *Wnt1-Cre* construct, we found significantly increased apoptosis in DRG and sympathetic neurons (George et al., 2013). In contrast, we did not find that *Ikbkap* deletion caused the death of embryonic CNS neurons. Moreover, we also examined cell death in the DRG of *Tuba1a-Cre*;*Ikbkap* CKO mice and found that deletion of *Ikbkap* in DRG neurons, rather than in their progenitor neural crest cells, did not induce their death during embryogenesis (*n*=3 embryos; TUNEL-positive cells/section: 3.7±0.2 in the control versus 2.6±0.4 in CKO; *P*<0.03; mean±s.e.m.). In support of this finding, we quantified the number of proprioceptive neurons (TrkC positive) in the DRG after the completion of the normal period of cell death (E17.5; Fig. S2f) and in the adult (*n*=4-5 adult 6-month-old mice; number of TrkC-positive neurons/section=15.1±2.8 in the control versus 15.4±1.2 in the CKO; *P*<0.92), and we found no difference between the CKO and littermate controls. It also appeared that the Ki67-positive progenitor population increased in the CKO at E14.5 (Fig. 6C,D), although future studies are needed to confirm this finding. Taken together, these data demonstrate that *Ikbkap* is required for normal cortical neurogenesis, which, in addition to increased apoptosis in the CKO adult, may contribute to the observed reduction in brain size. However*, Ikbkap* absence does not cause the death of CNS progenitors nor of post-mitotic CNS neurons during embryogenesis.

### Disorganization of the ventricular zone and of cilia in both embryonic progenitors and adult ependymal cells in the absence of *Ikbkap*

**Fig. 7.**
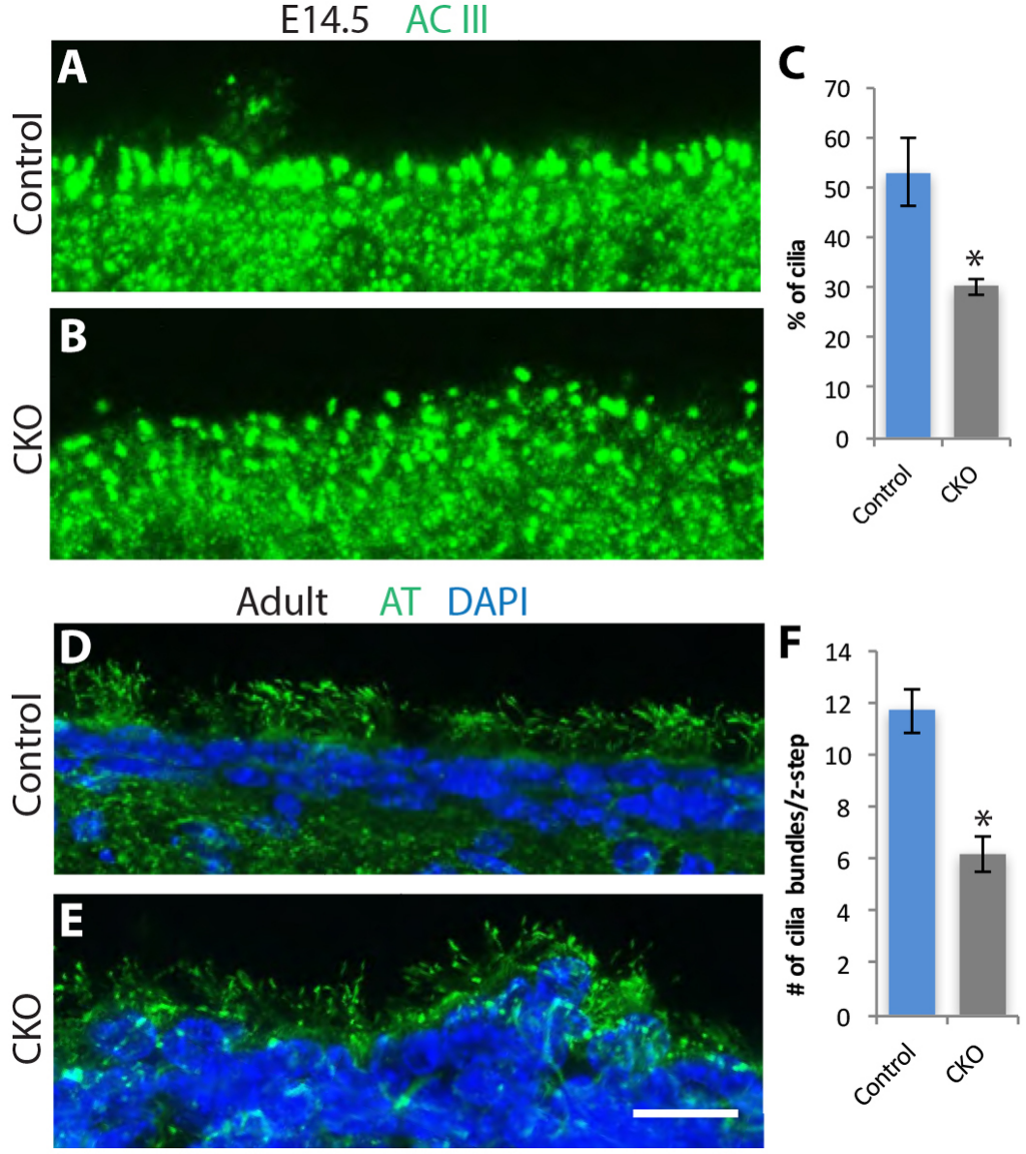
**Abnormal cilia in both the embryonic and adult lateral ventricles in the absence of *Ikbkap*.** (A-C). Embryonic E14.5 coronal sections were stained with antibodies to adenylate cyclase III (ACIII), and the number of cilia protruding into the lateral ventricles was quantified (*n*=3 embryos; *P<0.014; Student's *t*-test). (D-F). In the adult ependymal layer of the lateral ventricle, the number of ependymal cells extending tufts of cilia was significantly reduced in the CKO compared to in the control littermate. Note irregular organization of the ependymal cell layer in the CKO compared to the control. AT, acetylated tubulin. (*n*=3 brains; *P<0.007; Student's *t*-test). Scale bar: 15 μm (A,B); 20 μm (D,E).

Due to their critical role in signaling, disruptions in primary cilia in cortical progenitor cells result in severe perturbations in neurogenesis and in ventriculomegaly (Han and Alvarez-Buylla, 2010; Higginbotham et al., 2013; Paridaen et al., 2015). As described above, the lateral ventricles in both the embryonic and adult CKO brain were enlarged. Given the reductions in cortical and subcortical plate thickness measured in the embryonic *Ikbkap* CKO, and the fact that the cilia-like intraluminal buds extended by PKD2L1-positive neurons in the spinal cord were abnormal, we investigated whether the primary cilia on apical progenitor cells lining the lateral ventricles were intact during corticogenesis at E14.5 (Fig. 7A-C). While littermate control primary cilia were highly organized and regular in their shape and spacing, primary cilia in the CKO progenitor cells were more irregular in both shape and their localization, with significantly fewer primary cilia protruding into the ventricle in the CKO. In addition, cilia at the ventricle in the CKO were rounder and less elongated in shape than were those in their littermate controls. Also striking was the irregular organization of the apical progenitors; while the control littermate ventricular surface was smooth, the apical progenitor layer was relatively disorganized and uneven in the CKO (Figs 6 and 7).

Primary cilia are extended by apical progenitors or radial glial cells in the embryonic cortex. Postnatally, a subpopulation of radial glial cells give rise to the ependymal cells that form the epithelial lining of the adult lateral ventricle (Spassky et al., 2005). As ependymal cells mature, they extend tufts of motile cilia that beat, thereby moving cerebral spinal fluid through the ventricles, which helps maintain homeostasis of CSF; disruptions in them can lead to hydrocephalus and in addition, perturbations in the ionic composition of CSF and its transport (Banizs et al., 2005; Ibañez-Tallon et al., 2004; Sawamoto et al., 2006). Given the ventriculomegaly in the mutant embryonic and adult brain, and the disruptions in primary cilia in the embryonic cortex, we asked whether *Ikbkap* is required for normal adult ependymal cilia*.* Our data indicate that in the absence of *Ikbkap*, the number of ependymal cells extending tufts of cilia in the adult ventricle was significantly reduced (Fig. 7D-F). Furthermore, while all ependymal tufts were oriented in the same direction in the control, individual cilia and tufts were more randomly oriented in the CKO (Fig. 7D,E). As in the embryo, the *Ikbkap* CKO ventricular lining was also more irregular in morphology than that of littermate controls. While ventricles were enlarged in both the embryonic and adult CKO brains, the mice did not appear to be hydrocephalic.

### Behavioral testing reveals CKO mice have reduced anxiety, and impaired spatial learning and memory

**Fig. 8.**
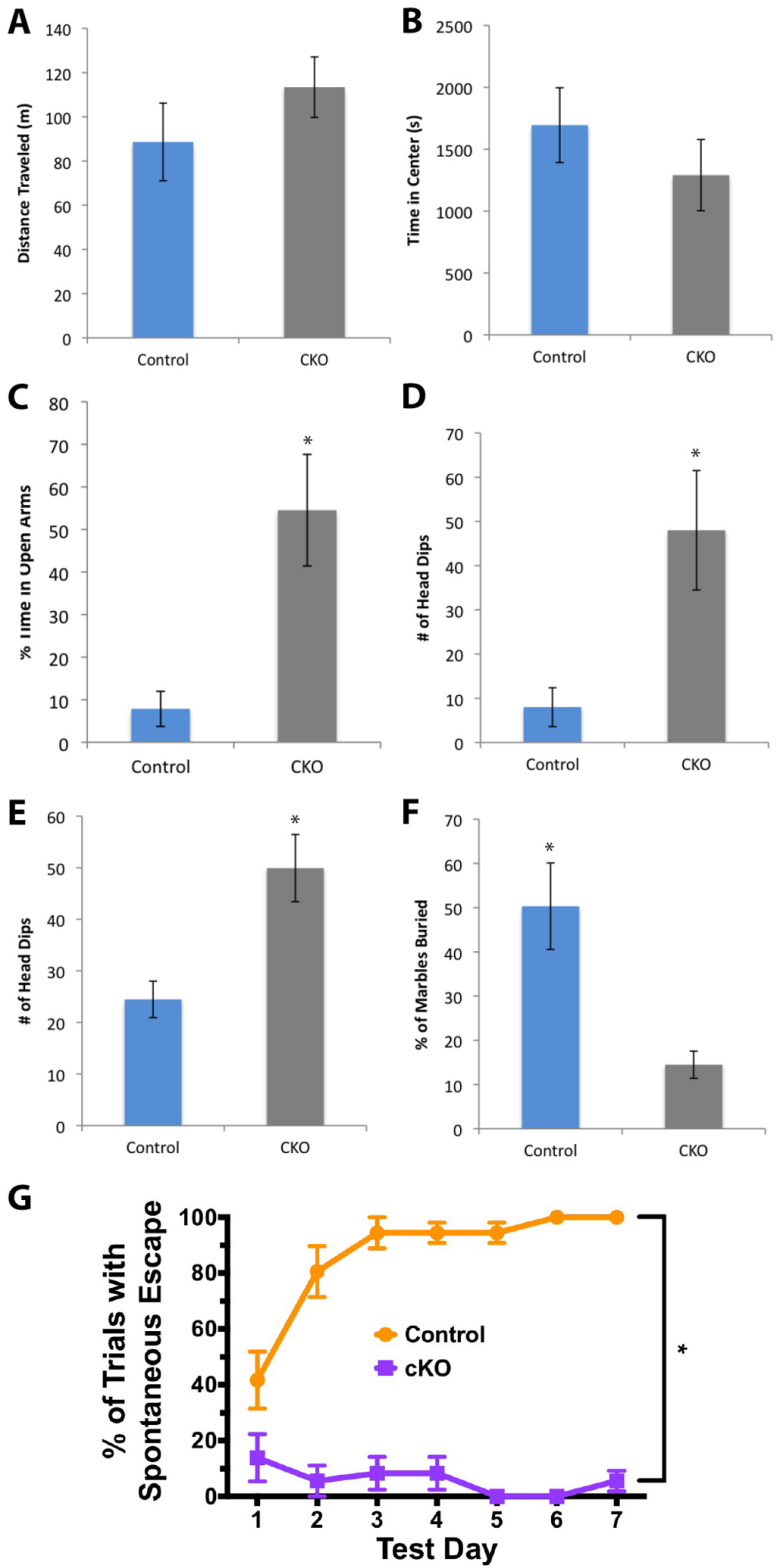
**CNS behavioral alterations in the absence of *Ikbkap*.** (A) CKO mice show no difference in locomotor activity compared to controls (*n*=8 per genotype; *P*>0.05) and (B) no difference in the amount of activity in the center versus at the edges (*P*>0.05). (C) On the EPM, CKO mice spend a greater proportion of time in the open arms (*n*=5 control, 6 CKO; *P<0.05) and (D) perform more head dips over the edge of the open arms (*n*=5 control, 6 CKO; *P<0.05). (E) A similar increase in head-dipping behavior is observed in the cliff avoidance paradigm (*n*=17 control, 13 CKO; **P<0.005). (F) In the marble burying task, CKO mice bury fewer marbles than controls (*n*=7 per genotype; *P<0.01). (G) In the Barnes maze, CKO mice fail to use spatial cues to learn the escape location over 7 days of training (*n*=9 per genotype; *P<0.0001). Error bars denote s.e.m. and *P-*values correspond to an unpaired Student's *t*-test.

In light of the significant reductions in several neuronal populations in the adult CNS of CKO mice, we investigated whether the mice exhibited alterations in behavior. All testing was done before mice showed signs of motor degeneration or weakness (less than 4 months old). Several behavioral paradigms were tested, including an open field activity test, the elevated plus maze (EPM), cliff avoidance reaction, marble burying behavior, and learning and memory. In the open field, CKO mice showed slightly increased locomotor activity (Fig. 8A) and activity in the center versus near the edges of the arena (Fig. 8B) compared to controls, but these differences were not significant. In the EPM, CKO mice spent a significantly greater proportion of time in the open arms compared to controls (Fig. 8C) and made significantly more head dips over the edges of the open arms (Fig. 8D). These two results indicate that CKO mice exhibit less anxiety compared to controls. We pursued other measures of spontaneous anxiety and found that in the cliff avoidance paradigm, CKO mice made significantly more head dips over the edge of the apparatus (Fig. 8E), and in the marble burying task, CKO mice buried significantly fewer marbles than their control counterparts (Fig. 8F). Finally, testing on the Barnes maze revealed that CKO mice exhibit a significant reduction in spatial learning and memory function (Fig. 8G).

## DISCUSSION

Because the devastation of the autonomic and sensory nervous systems dominate the clinical experience of patients with FD, much less attention has been devoted to CNS deficits. Yet CNS perturbations have been demonstrated in autopsy, pathology and imaging studies on FD patients, in EEG recordings, and as manifested in psychometric exams (Engel and Aring, 1945; Cohen and Solomon, 1955; Brown et al., 1964; Sak et al., 1967; Ochoa, 2003, 2004; Axelrod et al., 2010; Norcliffe-Kaufmann et al., 2016; Dietrich and Dragatsis, 2016). Our study is the first to directly investigate the CNS pathophysiology of an animal model for FD, and the results demonstrate that *Ikbkap* is required for the normal development, function and survival of several CNS subpopulations. Although *Ikbkap* is broadly expressed within the body, including in several internal organs (Mezey et al., 2003; Dietrich and Dragatsis, 2016), our work shows that deletion of *Ikbkap* solely in the nervous system is sufficient to recapitulate such classic FD features as kyphoscoliosis. Furthermore, our data are the first to demonstrate a requirement for *Ikbkap* in the maintenance of neuromuscular junction innervation and that its absence results in behavioral abnormalities, including cognitive dysfunction, thereby greatly expanding our understanding of the requirement for this gene in the CNS.

The ANS is part of both the CNS and PNS, and functions as an integrated network, processing sensory input and motor output to maintain homeostasis (Saper, 2002; Beissner et al., 2013). Key regulatory structures for the ANS include the hypothalamus and brainstem nuclei, including the nucleus tract solitarius (NTS) and dorsal motor nucleus of the vagus (DMV), and we show here that these structures express *Ikbkap*. The DMV is composed of pre-ganglionic parasympathetic neurons that regulate output from parasympathetic ganglia in the periphery, while sympathetic ganglia get their regulatory input from the intermediolateral cell column (IML) in the spinal cord. We demonstrate that in the absence of *Ikbkap*, neuronal number in both the DMV and IML is significantly reduced. Autopsy studies show that both of these cell populations are significantly reduced in FD patients (Engel and Aring, 1945; Cohen and Solomon, 1955; Pearson and Pytel, 1978). A decrease in DMV neurons is likely to lead to irregular control of cardiovascular reflexes, which could underlie the often sudden death of the CKO mice and the frequent cardiac arrest of FD patients. In fact, brainstem reflexes have been demonstrated to be abnormal in FD patients (Gutiérrez et al., 2015; Norcliffe-Kaufmann et al., 2016).

While clinical studies on FD patients have indicated that sympathetic output is intact, its regulation by faulty sensory input is the ultimate cause of a dysfunctional end organ activity (Norcliffe-Kaufmann et al., 2010, 2011). One of the most intriguing findings from the analysis reported here is the reduction in primary cilia, which act as critical signaling sensors during cortical development, and in adult ependymal motile cilia. Disruptions in primary cilia cause ciliopathies that affect brain development – including Joubert, Meckel–Gruber, orofaciodigital and Bardet–Biedl syndromes (Métin and Pedraza, 2014; Guo et al., 2015) – and impair kidney function, while disruptions in motile cilia can interfere with respiratory function in addition to CSF flow. It will be of interest to investigate whether cilia are disrupted in these non-neural areas given the organ function impairment in FD. The other key cilia-like sensory cells in the nervous system are the CSF-contacting neurons surrounding the central canal of the spinal cord, whose numbers were reduced and their morphology altered in the CKO mouse. These cells are pH sensors (Jalalvand et al., 2016) known to receive input from visceral pain receptors (Saper, 2002) and may mediate autonomic reflex responses (Willis et al., 1999). Their intraluminal endings strongly express PKD2Ll1-positive pH-regulated Ca^2+^ channels (DeCaen et al., 2013). These cells provide a homeostatic mechanism for regulating pH by stimulating motor activity (Böhm et al., 2016; Jalalvand et al., 2016). Thus loss of these endings could compromise the detection of pH changes in the CSF. FD patients do, in fact, have high levels of CO_2_ in their blood (Maayan et al., 2015), suggesting a breach in pH, O_2_ and/or CO_2_ detection and/or response to these factors in FD. Interestingly, baroreceptor function, another key source of afferent information to the CNS, is also compromised in FD patients (Norcliffe-Kaufmann et al., 2010).

Although multiple studies demonstrate the significant role that disruption of afferent sensory signaling plays in FD (Norcliffe-Kaufmann et al., 2010; Macefield et al., 2011, 2014; Norcliffe-Kaufmann and Kaufmann, 2012; Gutiérrez et al., 2015), there is also evidence for disruptions in motor neurons in FD – as manifested in decreases in motor nerve conduction velocity (Brown et al., 1967; Macefield et al., 2011) and in cell number (Dyck et al., 1978). Our work clearly demonstrates that not only is *Ikbkap* strongly expressed in spinal motor neurons but also that its loss leads to motor neuron death and progressive loss of muscle innervation. Gait ataxia is one of the predominant hallmarks of FD, and its severity increases as patients age. Patient recordings demonstrate a loss of type I and type II muscle spindle afferents (Macefield et al., 2011). Interestingly, we have now quantified proprioceptive neurons in both the *Ikbkap* CKO mouse model presented in this report and in a previous study using a different *Ikbkap* CKO model (George et al., 2013). In both cases, there was no reduction in the number of proprioceptive (TrkC positive) neurons (Fig. S2f). Given that FD patient ataxia progresses with age, we suggest that if the same mechanisms are operative in the human as in the mouse, then the loss of proprioceptors in patients most likely occurs as an indirect effect of the progressive demise of NMJ integrity and associated muscle weakness as demonstrated in this study or results from metabolic problems related to aging, as occurs with the progressive loss of retinal ganglion cells in the adult FD human and mouse retina (Ueki et al., 2016; Mendoza-Santiesteban et al., 2012, 2014). As such, proprioceptors may comprise a targetable population in order to prevent cell death.

In previous work, we have shown that DRG and sympathetic neurons that lack *Ikbkap* die by an activated caspase-3- and p53-mediated apoptotic mechanism (George et al., 2013), and here we show increased apoptotic death in the adult *Ikbkap* CKO brain (Fig. S2). It is of considerable interest to determine the mechanisms responsible for the neuronal death exhibited by both PNS and CNS neurons in FD. Given the progressive nature of motor weakness that develops in CKO adult mice, and the extensive human FD clinical data demonstrating progressive loss of neuronal populations, our data support a critical role for IKAP in maintaining adult neuronal survival. We have also recently demonstrated the progressive loss of retinal ganglion cells in adult mouse retina that lack *Ikbkap* (Ueki et al., 2016), which underlies the progressive optic neuropathy experienced by older FD patients (Mendoza-Santiesteban et al., 2012, 2014). A recent study has found that deletion of another Elongator subunit, *Elp3*, led to an elevation in endoplasmic reticulum (ER) stress and the unfolded protein response, which was attributed to reduced translation speed of codons read by U_34_-containing tRNAs that are dependent upon modification (Laguesse et al., 2015). Previous studies have shown that IKAP can bind to JNK and regulate the cytotoxic stress response (Holmberg et al., 2002). A major focus now is to identify the downstream survival pathways that are impacted by loss of *Ikbkap* function in order to identify potential therapeutic targets.

Our work reveals that not only is *Ikbkap* required in several adult neuronal CNS populations but that its absence perturbs CNS development. Cortical neurogenesis is reduced and ventricles are enlarged, which, although speculative at this point, may both result from inappropriate proliferation of apical progenitor cells and their failure to switch towards generating neurons indirectly, a transition that significantly amplifies neuronal numbers (Noctor et al., 2004). A recent study has shown that conditional deletion of *Elp3* inhibits the transition of apical progenitors to intermediate progenitors, thereby ultimately reducing neuronal levels (Laguesse et al., 2015). Similarly, in our previous analysis of *Ikbkap* CKO DRGs (George et al., 2013), we found that *Ikbkap* was required in the Pax3-positive TrkA progenitors and that its absence led to their premature differentiation and, consequently, a deficit in the number of TrkA-positive pain and temperature receptors. In the cortex, we found that the deficit in cortical neurons during embryonic development was not due to elevated cell death (Fig. S2) but rather to decreased neurogenesis (Fig. 6), the same findings as shown previously in the *Elp3* CKO (Laguesse et al., 2015). The perturbations to the cellular architecture of the ventricular zone and the disruption to primary cilia are also likely to contribute to the impaired cortical development that occurs in the absence of *Ikbkap*. Although a previous study has described a temporary delay in migration of nascent cortical neurons in the presence of reduced levels of *Elp3* mRNA (Creppe et al., 2009), in the *Ikbkap* CKO, cortical layering in the adult brain was in general normal (Fig. 3).

Approximately 80% of children with FD develop a spinal deformity – scoliosis and/or kyphosis – by their early 20s (Bar-On et al., 2000; Hayek et al., 2000). The pathophysiological mechanisms responsible for kyphosis and scoliosis in children are varied and include disc degeneration, osteoporosis, connective tissue disorders and neuromuscular weakness, such as occurs in poliomyelitis or Duchenne's Muscular Dystrophy. The fact that the majority of mice with CNS-specific *Ikbkap* ablation developed kyphosis suggests that its cause in FD may result directly from reduced levels of IKAP in CNS neurons, rather than from skeletal or connective tissue disruptions. We hypothesize that depletion of IKAP in spinal motor neurons impairs muscle innervation, thereby weakening the erector spinae muscles that are required for maintaining a straight spinal cord column. Thus, mitigation of spinal motor neuron death could potentially thwart the development of the spinal curvature abnormalities in FD. Interestingly, allelic variation of *ELP3* is associated with the neuromuscular disorder amyotrophic lateral sclerosis (ALS) (Simpson et al., 2009). Our work suggests that compromised Elongator complex function, via mutation in either *IKBKAP* or *ELP3*, can both converge to cause the death of spinal motor neurons and associated sequelae. Given the variation in splicing efficiency in FD cells, motor neurons may be relatively spared in patients relative to those in the *Ikbkap* CKO mouse.

One of the most surprising findings from this study is the disruption in normal behavior of CKO mice. While the ANS is known to involve cortical and subcortical circuits, our understanding of how these regions are integrated to affect behavior (Benarroch, 1993; Verberne and Owens, 1998; Saper, 2002; Beissner et al., 2013) are not fully understood. With the exception of thigmotaxis, in which there was no significant difference, CKO mice display reduced anxiety behaviors, including more time in the open arms of the EPM (Espejo, 1997; Sorregotti et al., 2013), more head dips over the apparatus edge during the cliff avoidance task (Soares et al., 2013) and apparent indifference to foreign objects in the marble burying paradigm (Dixit et al., 2014; Kedia and Chattarji, 2014). Consistent with the decreased anxiety exhibited by the CKO mice, our morphometric analysis also revealed that the lateral amygdaloid nucleus was reduced in the CKO brain. These data are quite surprising given that FD patients sometimes exhibit a heightened anxiety state (Clayson et al., 1980; Norcliffe-Kaufmann et al., 2016). These differences could be due to different expression patterns and/or loss of function of *Ikbkap* in this mouse model versus human. Although speculative, the anxiety manifested in human patients may be in response to social cues and an awareness of their serious life-threating medical condition. Alternatively, since the sensory and sympathetic nervous system in our mouse model was less impacted than that in most patients, the heightened anxiety levels in human FD may result primarily from disruptions in their peripheral autonomic activity. In addition to reduced anxiety, CKO mice display a significant spatial learning and memory deficit, as measured on the Barnes maze. The role of the hippocampus in learning and memory is well established (Jarrard, 1993; Sprick, 1995); however, our data show that *Ikbkap* was not deleted from the hippocampus of CKO animals, suggesting that some other component of the learning and memory circuitry is not functioning properly. Further research aimed at characterizing the emotional and cognitive phenotype of this novel model of FD is needed to clarify the impact of altered *Ikbkap* in the CNS.

Like Rett syndrome, which is marked by both an intellectual disability and a dysautonomia, the accruing data on FD indicates that FD should not be classified solely as an HSAN (Dietrich and Dragatsis, 2016), nor should HSAN IV and HSAN V, which in addition to the peripheral dysfunction observed, are marked by intellectual disability and/or dementia. This study demonstrates that not only is *Ikbkap* broadly expressed in the CNS, beyond simply ANS nuclei, but that its loss leads to alterations to many CNS structures with effects on cognitive function. Finally, this study should be considered in the broader context of the requirement for the six-subunit Elongator complex in the CNS. Variants in the *ELP2* gene are associated with neurodevelopmental (including intellectual) disability (Cohen et al., 2015; Franic et al., 2015) while *ELP3* variants are associated with ALS (Simpson et al., 2009; Kwee et al., 2012), and loss of *ELP4* can cause Rolandic epilepsy syndrome and intellectual disability (Strug et al., 2009; Nguyen et al., 2010; Gkampeta et al., 2014; Reinthaler et al., 2014; van Blitterswijk et al., 2014; Addis et al., 2015). This study adds to the growing understanding of the critical function for the Elongator complex in the CNS by revealing the repercussions of *Ikbkap* loss in the CNS. Taken together, these findings point to the need for further studies on the function of Elongator in the nervous system; important questions remain, such as whether the individual Elongator subunits exclusively function together as the Elongator complex, and/or whether they also function as components of other enzymatic complexes and/or have independent functions, and why effects of the loss of the different Elongator subunits manifest in distinct neurological disorders.

## MATERIALS AND METHODS

### Mice

All experiments conform to all relevant institutional and national animal welfare laws, guidelines and policies, as did the care and use of experimental animals. *Ikbkap^tm1a^^(KOMP)Wtsi−^* ‘knockout first’ mice containing an *frt*-flanked *LacZ Ikbkap* reporter that disrupts IKAP expression before the *LoxP* flanked 4th exon of *Ikbkap* (Fig. 1A) were obtained from the International Knockout Mouse Consortium. This *Ikbkap* knockout allele was found to be homozygous lethal by E10, but *Ikbkap* expression could be rescued by removal of the β-gal cassette via flippase-mediated recombination. The new allele contains *LoxP* sites flanking the *Ikbkap* coding sequence, providing the opportunity to generate a CKO. Removal of the floxed region via Cre recombinase is predicted to yield a truncated product that is subject to non-sense mediated decay. We crossed homozygous *Ikbkap* CKO (*I^C/C^*) mice to hemizygous *Alpha-tubulin 1a-Cre* (*Tuba1a-Cre*) mice (a gift from Dr Lino Tessarollo, National Cancer Institute, Frederick, MD) that also carried one copy of the *Ikbkap* CKO allele expecting a 1:1:1:1 ratio of the following genotypes: (1) *Tuba1a-Cre;I^C/C^*, (2) *Tuba1a-Cre;I^+/C^*, (3) *I^C/C^* and (4) *I^+/C^* . However, out of 116 mice, 12 were class 1, 47 were class 2, 48 were class 3, and 9 were class 4 (chi square analysis between expected and observed ratios gives a *P*-value of ≤0.001). One possible explanation for this altered segregation ratio is that the *Tuba1a-Cre* gene is linked to the *Ikbkap* locus. In this case, the wild-type *Ikbkap* allele in the original *Cre* mouse would tend to segregate with the *Cre* gene. Depending on the degree of linkage, *Tuba1a-Cre;I^C^* and *I^+^* gametes would be rare, reducing the number of progeny in the *Tuba1a-Cre;I^C/C^* and the *I^+/C^* classes. To test this hypothesis, we crossed CKO males (*Tuba1a-Cre;I^C/C^*) and *I^C/C^* females, and saw the expected 1:1 Mendelian ratio of CKO (*Tuba1a-Cre;I^C/C^*) and control (*I^C/C^*) mice (65:64, *P*-value≥0.93), confirming linkage between the *Tuba1a-Cre* and *Ikbkap* loci. For all analyses, *Tuba1a-Cre;I^C/C^* mice were used as experimental and *I^C/C^* littermates were used as controls. These genotypes were produced by crossing *Tuba1a-Cre;I^C/C^* CKO males with *I^C/C^* females, which generated experimental and control mice at a 1:1 ratio. All genotyping was performed via PCR (Fig. 1B). *Ikbkap* CKO and wild-type alleles were distinguished using the following primers: forward, 5′-GCACCTTCACTCCTCAGCAT-3′; and reverse, 5′-AGTAGGGCCAGGAGAGAACC-3′. Recognition sites for these primers flank both the remaining FRT site and one LoxP site (positions shown in Fig. 1A), yielding a band of 498 bp for the CKO allele and of 384 bp for the wild-type allele. Although Cre-mediated recombination removes the reverse primer site in the nervous system, the presence of DNA from other cell types allows for detection of the CKO allele in mutant mice. The presence of the *Tuba1a-Cre* gene was detected using primers: forward, 5′-TGCTCTTTCACTGGTTATGCGG-3′; and reverse, 5-TTGCCCCTGTTTCACTATCCAG-3 (general Cre primers). Mice bearing the *ROSAmT-mG* allele (stock no. 007576) were purchased from the Jackson Laboratory. In its non-recombined state, *ROSAmT-mG* provides red fluorescence in all cells. Following Cre-dependent recombination, the fluorescence is converted from red to green.

### Immunohistochemistry and cell quantification

Embryonic sections were stained as described with Tuj1 and DAPI or for β-gal (George et al., 2013). Antibodies against the following proteins were also used: Olig 2 (rabbit, 1:1000; Millipore, #AB15328), GFP (chicken, 1:2000; Abcam, #ab13970), caspase-3 (cleaved, rabbit, 1:250; Cell Signaling, #9664), Substance P (Guinea pig, 1:1000; Abcam, #ab10353), CGRP (Rabbit, 1:400; Calbiochem, #PC205L), TrkC (goat, 1:2000; R&D Systems, #AF1404), Ki-67 (rabbit, 1:400; Sigma, #SAB5500134), adenyl cyclase III (rabbit, 1:250; Santa Cruz Biotechnology, sc-588), acetylated tubulin (mouse, 1:300, Sigma, #T745), β-catenin (rabbit, 1:2000; Sigma, #C2206). The In Situ Cell Death Detection Kit (TMR red, Roche, #12156792910) was used to detect apoptotic cells, and EdU staining was performed according to the manufacturer's instructions (Click-iT EdU Alexa Fluor 647 Imaging Kit, #C10337, Thermo Fisher Scientific). Adult motor neurons in the brain stem and spinal cord were immunolabeled with antibodies to ChAT (goat polyclonal, 1:150; Millipore #AB144P). Cryosections from adult mice were stained as described previously in George et al. (2013) or if antigen retrieval was required, were immersed in 80-90°C citrate buffer (10 mM sodium citrate, 0.05% Tween 20, pH 6.0) followed by incubation at room temperature for 1 h before immunolabeling. Positive ChAT cells were counted in the ventral horn of spinal cord gray matter from lumbar levels 1-6; three mice per time point per treatment were analyzed. To quantify neurons in the intermediolateral cell column in the thoracic spinal cord, sections (T7-T13) were stained with a rabbit polyclonal against nNOS (Immunostar #24287, 1:4000). To quantify ChAT-positive neurons in the dorsal motor nucleus of the Vagus (DMV), 20-μm sections were photographed using a Leica TCS SP8 confocal microscope, and *z*-stack images were obtained using Leica Application Suite Advanced Fluorescence software. All sections containing the DMV were photographed, and the number of ChAT-positive cells in each section was recorded. The number of cells in three sections where the DMV was largest was averaged, and used to calculate averages of mutant and control groups. Cell numbers were compared using an unpaired two-tail *t*-test. PKD2L1-positive (AB 9084, Millipore; 1:700) cells in thoracic spinal cord (T6-T13) of control and CKO mice, and positive endings that protruded into inside the central canal were quantified. Two types of ending interactions were tabulated: no direct contacts among the intraluminal buds or multiple PKD2L1-positive endings were juxtaposed inside the central canal.

### Cilia analysis

Primary cilia were immunolabeled and imaged as described above and analyzed using ImageJ. In the adult, ependymal cilia were stained with antibodies to acetylated tubulin and β-catenin as described above, and the number of cilia bundles per *z*-step were determined; a bundle was defined as being composed of at least two cilia with both cilia visible in their entirety. In the embryo, cilia shape was also quantified using National Institutes of Health (NIH) ImageJ software, and the morphology was compared using an unpaired *t*-test.

### Western blots and densitometry

Brains were harvested from mice euthanized via CO_2_ inhalation and immediately placed on an ice-cold metal platform and the frontal cortex, hippocampus and striatum were dissected, flash-frozen and stored at −80°C. Frozen tissue was suspended in 1 ml of homogenization buffer [130 mM NaCl, 1.3 mM KCl, 2.2 mM CaCl_2_, 1.2 mM MgSO_4_, 1.2 mM KH_2_PO_4_, 10 mM Hepes (pH 7.4), 10 mM glucose and 0.32 M sucrose]. Protein concentrations of all samples were determined by using a bicinchroninic acid (BCA) protein assay (Thermo Fisher Scientific). Equal amounts of protein were incubated with Laemmli sample buffer for 1 h at room temperature and analyzed by SDS-PAGE and immunoblotting. IKAP protein was visualized in samples blotted to a PVDF membrane (Bio-Rad) with a rabbit anti-IKAP antibody (1:500; AS-54494, Anaspec), and GAPDH was visualized using a rabbit anti-GAPDH antibody (Millipore, #ABS16; 1:10,000) as a loading control. Horseradish peroxidase (HRP)-conjugated secondary antibody (Southern Biotech) was detected using Pierce ECL Western Blotting Substrate (Thermo Fisher Scientific) and exposure to B-Plus Full Blue X-ray film (Phenix Research Products). Multiple exposures were obtained to ensure linearity of band detection. Western blots were quantified using NIH ImageJ software and analyzed via unpaired *t*-test for each brain region (Graphpad Prism 7.0a).

### CNS morphology

Mice were euthanized using a method approved by the Institutional Animal Care and Use Program. Mice were transcardially perfused with 35 ml of 1× PBS followed by 35 ml of ice-cold 4% paraformaldehyde solution. Brains were dissected and placed into 4% paraformaldehyde and fixed for 24 h at 4°C. Tissue was then stored in 30% sucrose in 0.1 M phosphate buffer solution and stored at 4°C until sectioning. Brains from non-perfused mice were dissected out and fixed as above. Brains were then embedded in Optimal Cutting Temperature (OCT) gel blocks and flash frozen, then sectioned coronally at 20 μm on a cryostat or using a sliding microtome. Sections were placed into cryoprotectant solution and stored at −20°C until used. Sections were stained with Cresyl Violet, staining all Nissl bodies within the cells. For quantitative analysis, images were loaded into OsiriX version 5.6 image-processing software. Regions of interest (ROIs) for each brain section were outlined, and the hemisphere area was calculated by the program in arbitrary units. An area measure for the corresponding hemisphere for each ROI was calculated as well. For each measurement, three serial sections were chosen 40 μm apart at identical coronal levels for each animal. Lateral ventricles: the boundaries for the lateral ventricles were drawn at the level of the fimbria. Corpus callosum: the boundaries for the corpus callosum were drawn from the midline just dorsal to the dorsal fornix and directly ventral to the cingulate bundle. The fiber bundles were then traced between the cortex and caudate to level of the rhinal fissure. Lateral amygdaloid nucleus: the lateral amygdaloid nucleus was traced from the split of the external capsule and border of the amygdala to the basolateral amygdalar nuclei. Thalamus: the thalamus was outlined using landmarks in the Allen atlas. Neocortex: the full thickness of the cortex (posterior to the crossing of the corpus callosum) was measured from the medial wall of the hemisphere to the rhinal fissure. Hippocampus: the hippocampus was measured from the area dorsal to the third ventricle, and immediately ventral to the dorsal fornix and corpus callosum. The structure was traced following the lateral edges of the stratum oriens of cornu ammonis (CA) fields 1, 2 and 3 and continued along the ventral edge of the dentate gyrus.

### Data analysis

Ten control and 11 mutant mice were used for each ROI. Each structural measure was divided by a measurement of the corresponding hemisphere to generate a ratio. This controlled for overall size differences between brains. The ratios were averaged for each brain. The control values for a given ROI were compared to the corresponding values for mutant animals using an unpaired two-tail *t*-test, with an α level less than 0.05 considered significant. Measures of s.d. and s.e.m. were also recorded.

### Neuromuscular junction staining and image analysis

Anterior tibialis muscles and erector-spinae muscle were dissected in PBS, and all connective tissue removed. Tissue was fixed for 20 min in 4% paraformaldehyde at room temperature, washed and then incubated for 15 min in α-bungarotoxin (Molecular Probes, #B160; 1 μg/ml) at room temperature, followed by PBS washes, and a 10 min incubation in −20°C methanol. After a 1 h block in PBS+2.0% BSA+0.7% Triton X-100+0.1% sodium azide, tissue was incubated for 48 h in primary antibodies at room temperature. Primary antibodies used were: monoclonal antibodies 2H3 (1:300) and SV2 (1:25) both from Developmental Hybridoma Bank, and a rabbit polyclonal S100 antibody (Z0311, Dako; 1:400). After washing, tissue was incubated for 48 h in secondary antibodies (Life Technologies: goat anti-mouse conjugated to Alexa Fluor 488; 1:1000; goat anti-rabbit conjugated to Alexa Fluor 633; 1:1000) rocking at room temperature, washed and then filleted for mounting and analysis. All imaging was done on a Leica TCS Sp8, with a 63× objective. *z*-stacks were analyzed using ImageJ. The number of α-bungarotoxin-positive receptor plaques that were innervated was determined, and NMJs were categorized based on whether they were fully or partially innervated and to what extent the junctional regions were intact with distinct edges versus frayed and diffuse borders.

### Behavior analysis

The behavioral phenotypes of CKO and age-matched control mice were evaluated in the following screens. All behavioral data were collected using an automated tracking system (AnyMaze, Stoelting) and blinded coding of relevant behaviors, when necessary. For each study, the apparatus was wiped with Clidox and dried between subjects. Data from each experimental paradigm were analyzed using unpaired Student's *t*-tests. All mice were 2-3 months old when tested, except where noted.

#### Neurological screen

In order to assess the overall health, reflexes and basic sensorimotor function, mice were tested using a modified version of the Irwin test battery (Mergy et al., 2014) and the SHIRPA protocol (Rogers et al., 1997). Toe pinch was performed using forceps; a jerk reflex was assessed, with a positive jerk reflex considered normal. For testing of heat sensitivity, each mouse was placed on a pre-warmed 55°C hot plate for up to 5 s and monitored for signs of discomfort (licking of paws, fanning of fore- or hind paws, jumping in the air or off of the hot plate). Hind limb clasping was assayed by suspending each mouse by its tail to a height of approximately 30 cm. Hind limb clasping was scored as: no clasping/hind limbs spread laterally (score=0), partial clasping (score=1) and full clasping (score=2).

#### Locomotor activity and thigmotaxis

Locomotor activity in the open field was measured in a 40×40×40-cm chamber. Mice were habituated to the testing room (30 min), then recorded for 60 min. Cumulative activity was analyzed using an unpaired Student’s *t*-test (GraphPad Prism 6.0). To measure thigmotaxis, activity was assessed in a 5-cm wide area immediately inside the apparatus walls (edge zone) versus the remaining interior of the testing arena (center zone).

#### Elevated plus maze and cliff avoidance

Mice were evaluated on the EPM, as previously described (Rodgers and Cole, 1993). After habituation to the testing room (30 min), individual mice were placed onto an open arm, facing the central platform, and recorded for 5 min. Head-dipping behavior was scored by an observer blind to the mice genotype. Mice were 1.5 months old when tested. Cliff avoidance was assayed as previously described (Yamashita et al., 2013).

#### Marble burying

Marble burying was assayed per a previously published protocol (Deacon, 2006). Briefly, mice were individually placed into an arena containing 5 cm of standard bedding, on top of which 20 black glass marbles were arranged in a 4×5 marble array. The mouse was allowed to explore the arena undisturbed for 30 min, during which the mouse's activity was recorded. The number of marbles visible after the task was analyzed using ImageJ software (NIH, Bethesda, MD) and reported as the percent of marbles buried.

#### Barnes maze

The Barnes maze was used to assess spatial learning and memory (Inman-Wood et al., 2000; Williams et al., 2003; Sunyer et al., 2007; Patil et al., 2009). The maze consisted of a round white acrylic plastic platform 90 cm in diameter with twenty 5-cm holes evenly spaced around the perimeter. The platform was elevated 75 cm above the floor. All of the holes can be covered to prevent a mouse from passing through, and one hole has an escape chamber (10.5×16×7 cm) affixed under the platform so that the mouse can escape from the open platform. On the walls of the testing room, spatial cues [white square signs containing 30-cm black shapes (circle, square and triangle)] were present. During the acclimation phase, the holes in the platform were covered except for the escape location, and the escape chamber was oriented towards one of the spatial cues. Each mouse was placed under a start chamber (7.5 cm diameter×7.5 cm high) in the center of the platform for 10 s. The start chamber was then removed, and noxious stimuli were presented: illumination [bright lights (ambient light=337 lux, bright light=511 lux)] and white noise (85 dB) played. The mouse was allowed to explore the platform for up to 3 min; if the mouse spontaneously entered the escape chamber, the noxious stimuli (bright lights and white noise) were turned off and the mouse was left in the escape chamber for 1 min; if the mouse did not spontaneously enter the escape chamber during the 3-min trial, the mouse was gently guided to the escape chamber, at which time the noxious stimuli were stopped. For the acquisition phase, the same set-up was used, except that the holes in the platform were uncovered. Each mouse received four trials per day, with a 15-min inter-trial interval. The escape chamber was located in the same position for all trials for each mouse, but counterbalanced across genotypes. Mice were trained to learn the location of the escape chamber for seven consecutive days. Following acquisition, mice underwent a probe trial. The escape chamber was removed and the hole leading to the escape chamber was blocked. Each mouse was allowed to explore the platform for 90 s. Recordings were analyzed to determine the percentage of spontaneous escape trials during the acquisition phase.
